# Methyl 4-(4-fluoro­phen­yl)-6-isopropyl-2-[*N*-methyl-*N*-(methylsulfonyl)amino]­pyrimidine-5-carboxyl­ate

**DOI:** 10.1107/S1600536808013524

**Published:** 2008-05-21

**Authors:** Wei He, Dong-Ling Yang, Yong-Tao Cui, Ye-Ming Xu, Cheng Guo

**Affiliations:** a Department of Applied Chemistry, College of Science, Nanjing University of Technolgy, Xinmofan Road No. 5, Nanjing 210009, People’s Republic of China; b Nanjing Frochem Tech. Co. Ltd, Xinmofan Road No. 36, Nanjing 210009, People’s Republic of China

## Abstract

In the mol­ecule of the title compound, C_17_H_20_FN_3_O_4_S, the pyrimidine and benzene rings are oriented at a dihedral angle of 35.59 (3)°. Intra­molecular C—H⋯N and C—H⋯O hydrogen bonds result in the formation of one five- and two six-membered non-planar rings. One of the six-membered rings adopts a chair conformation, while the other six-membered ring and the five-membered ring exhibit envelope conformations with O and N atoms displaced by 0.837 (3) and 0.152 (3) Å, respectively from the planes of the other ring atoms. In the crystal structure, inter­molecular C—H⋯F hydrogen bonds link the mol­ecules into infinite chains.

## Related literature

For ring puckering parameters, see: Cremer & Pople (1975 ▶).
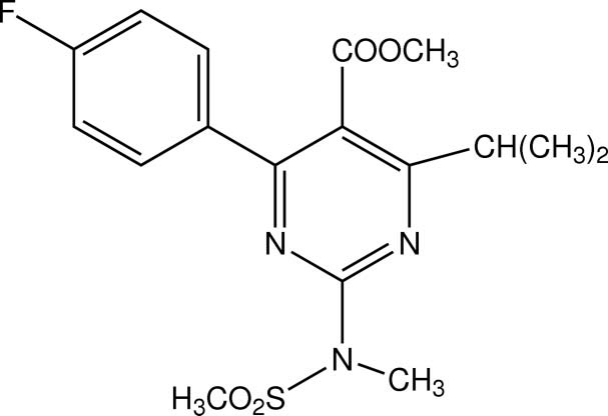


## Experimental

### 

#### Crystal data

C_17_H_20_FN_3_O_4_S
*M* *_r_* = 381.42
Orthorhombic, 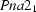

*a* = 9.886 (2) Å
*b* = 9.988 (2) Å
*c* = 18.819 (4) Å
*V* = 1858.2 (7) Å^3^
*Z* = 4
Mo *K*α radiation
μ = 0.21 mm^−1^
*T* = 294 (2) K
0.30 × 0.20 × 0.10 mm

#### Data collection

Enraf–Nonius CAD-4 diffractometer
Absorption correction: ψ scan (North *et al.*, 1968 ▶) *T*_min_ = 0.939, *T*_max_ = 0.979
3641 measured reflections
3641 independent reflections
2501 reflections with *I* > 2σ(*I*)
3 standard reflections frequency: 120 min intensity decay: none

#### Refinement

*R*[*F* ^2^ > 2σ(*F* ^2^)] = 0.075
*wR*(*F* ^2^) = 0.182
*S* = 1.04
3641 reflections
235 parameters
1 restraint
H-atom parameters constrained
Δρ_max_ = 0.23 e Å^−3^
Δρ_min_ = −0.25 e Å^−3^
Absolute structure: Flack (1983 ▶), 1755 Friedel pairs
Flack parameter: 0.14 (16)

### 

Data collection: *CAD-4 Software* (Enraf–Nonius, 1989 ▶); cell refinement: *CAD-4 Software*; data reduction: *XCAD4* (Harms & Wocadlo, 1995 ▶); program(s) used to solve structure: *SHELXS97* (Sheldrick, 2008 ▶); program(s) used to refine structure: *SHELXL97* (Sheldrick, 2008 ▶); molecular graphics: *PLATON* (Spek, 2003 ▶); software used to prepare material for publication: *SHELXTL* (Sheldrick, 2008 ▶).

## Supplementary Material

Crystal structure: contains datablocks D, I. DOI: 10.1107/S1600536808013524/hk2456sup1.cif

Structure factors: contains datablocks I. DOI: 10.1107/S1600536808013524/hk2456Isup2.hkl

Additional supplementary materials:  crystallographic information; 3D view; checkCIF report

## Figures and Tables

**Table 1:** Hydrogen-bond geometry (Å, °)

*D*—H⋯*A*	*D*—H	H⋯*A*	*D*⋯*A*	*D*—H⋯*A*
C3—H3*B*⋯O1	0.98	2.52	3.181 (8)	125
C10—H10*B*⋯N1	0.96	2.56	3.148 (7)	120
C11—H11*A*⋯N2	0.96	2.23	2.697 (7)	109
C11—H11*C*⋯F^i^	0.96	2.34	3.302 (8)	177

Symmetry code: (i) 
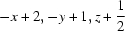
.

**Fig. 1.**
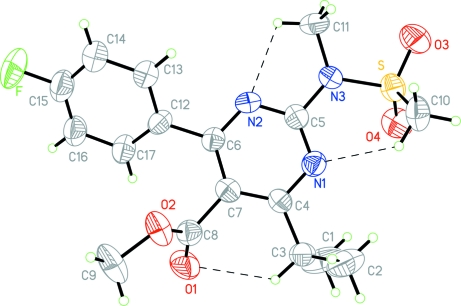
The molecular structure of the title molecule, with the atom-numbering scheme. Displacement ellipsoids are drawn at the 30% probability level. Hydrogen bonds are shown as dashed lines.

**Fig. 2.**
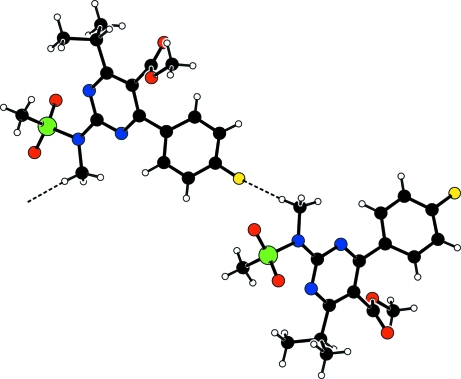
A partial packing diagram for (I). Hydrogen bonds are shown as dashed lines.

## supplementary crystallographic information

### Comment

Some derivatives of pyrimidine are important chemical materials. We report herein the crystal structure of the title compound, (I).

In the molecule of (I), (Fig. 1), rings A (N1/N2/C4-C7) and B (C12-C17) are, of course, planar, and the dihedral angle between them is A/B = 35.59 (3)°. The intramolecular C-H···N and C-H···O hydrogen bonds (Table 1) result in the formation of one five- and two six-membered non-planar rings: C (N2/N3/C5/C11/H11A), D (S/N1/N3/C5/C10/H10B) and E (O1/C3/C4/C7/C8/H3B), respectively. Ring D adopts chair [φ = -40.04 (2)° and θ = 134.72 (3)°] conformation, having total puckering amplitude, Q_T_, of 1.188 (3) Å (Cremer & Pople, 1975). Rings C and E have envelope conformations with nitrogen and oxygen atoms displaced by 0.152 (3) Å and 0.837 (3) Å from the planes of the other ring atoms, respectively.

In the crystal structure, intermolecular C-H···F hydrogen bonds (Table 1) link the molecules into infinite chains (Fig. 2), in which they may be effective in the stabilization of the structure.

### Experimental

For the preparation of the title compound, sodium salt of N-methyl methane sulphonamide (106 g, 631.00 mmol) and methyl 4-(4-fluorophenyl)-6-isopropyl- 2-methyl sulfonylpyrimidine-5-carboxylate (100 g, 284.06 mmol) were added to DMF (1000 ml) in a round bottom flask, and then stirred for 1 h at 303 K. After completion of the reaction, demineralized water (1000 ml) was added and stirred for 1 h. The mixture was filtered, washed with water, and then dried (yield; 95%). Crystals of (I) suitable for X-ray analysis were obtained by slow evaporation of an ethanol solution.

### Refinement

H atoms were positioned geometrically, with C-H = 0.93, 0.98 and 0.96 Å for aromatic, methine and methyl H, respectively, and constrained to ride on their parent atoms with U_iso_(H) = xU_eq_(C), where x = 1.5 for methyl H, and x = 1.2 for all other H atoms.

### Crystal data

**Table:**

C_17_H_20_FN_3_O_4_S	*F*_000_ = 800
*M**_r_* = 381.42	*D*_x_ = 1.363 Mg m^−^^3^
Orthorhombic, *P**n**a*2_1_	Mo *K*α radiation λ = 0.71073 Å
Hall symbol: P 2c -2n	Cell parameters from 25 reflections
*a* = 9.886 (2) Å	θ = 9–13º
*b* = 9.988 (2) Å	µ = 0.21 mm^−^^1^
*c* = 18.819 (4) Å	*T* = 294 (2) K
*V* = 1858.2 (7) Å^3^	Block, colorless
*Z* = 4	0.30 × 0.20 × 0.10 mm

### Data collection

**Table:**

Enraf–Nonius CAD-4 diffractometer	*R*_int_ = 0.062
Radiation source: fine-focus sealed tube	θ_max_ = 26.0º
Monochromator: graphite	θ_min_ = 2.2º
*T* = 294(2) K	*h* = 0→12
ω/2θ scans	*k* = 0→12
Absorption correction: ψ scan(North *et al.*, 1968)	*l* = −23→23
*T*_min_ = 0.939, *T*_max_ = 0.979	3 standard reflections
3641 measured reflections	every 120 min
3641 independent reflections	intensity decay: none
2501 reflections with *I* > 2σ(*I*)	

### Refinement

**Table:**

Refinement on *F*^2^	Hydrogen site location: inferred from neighbouring sites
Least-squares matrix: full	H-atom parameters constrained
*R*[*F*^2^ > 2σ(*F*^2^)] = 0.075	*w* = 1/[σ^2^(*F*_o_^2^) + (0.070*P*)^2^ + 2.*P*] where *P* = (*F*_o_^2^ + 2*F*_c_^2^)/3
*wR*(*F*^2^) = 0.182	(Δ/σ)_max_ < 0.001
*S* = 1.04	Δρ_max_ = 0.23 e Å^−^^3^
3641 reflections	Δρ_min_ = −0.25 e Å^−^^3^
235 parameters	Extinction correction: none
1 restraint	Absolute structure: Flack (1983), 1755 Friedel pairs
Primary atom site location: structure-invariant direct methods	Flack parameter: 0.14 (16)
Secondary atom site location: difference Fourier map	

### Special details

**Table:**

Geometry. All e.s.d.'s (except the e.s.d. in the dihedral angle between two l.s. planes) are estimated using the full covariance matrix. The cell e.s.d.'s are taken into account individually in the estimation of e.s.d.'s in distances, angles and torsion angles; correlations between e.s.d.'s in cell parameters are only used when they are defined by crystal symmetry. An approximate (isotropic) treatment of cell e.s.d.'s is used for estimating e.s.d.'s involving l.s. planes.
Refinement. Refinement of F^2^ against ALL reflections. The weighted R-factor wR and goodness of fit S are based on F^2^, conventional R-factors R are based on F, with F set to zero for negative F^2^. The threshold expression of F^2^ > 2sigma(F^2^) is used only for calculating R-factors(gt) etc. and is not relevant to the choice of reflections for refinement. R-factors based on F^2^ are statistically about twice as large as those based on F, and R- factors based on ALL data will be even larger.

### Fractional atomic coordinates and isotropic or equivalent isotropic displacement parameters (Å^2^)

**Table:**

	*x*	*y*	*z*	*U*_iso_*/*U*_eq_	
S	0.55906 (13)	0.70239 (15)	0.79080 (8)	0.0491 (4)	
O1	0.8512 (5)	1.1302 (5)	0.5173 (3)	0.0717 (13)	
O2	1.0367 (4)	1.0166 (5)	0.5494 (2)	0.0611 (12)	
O3	0.5199 (5)	0.5777 (4)	0.8218 (3)	0.0728 (14)	
O4	0.4566 (4)	0.7948 (5)	0.7692 (2)	0.0672 (14)	
N1	0.6898 (5)	0.8871 (5)	0.6925 (2)	0.0463 (12)	
N2	0.7773 (4)	0.7113 (4)	0.6207 (2)	0.0396 (10)	
N3	0.6541 (5)	0.6626 (4)	0.7201 (2)	0.0424 (10)	
F	1.1194 (5)	0.5725 (5)	0.3523 (2)	0.1013 (16)	
C1	0.5763 (7)	1.1558 (7)	0.6613 (5)	0.088 (3)	
H1B	0.5538	1.1425	0.6121	0.132*	
H1C	0.5598	1.2474	0.6740	0.132*	
H1D	0.5216	1.0982	0.6902	0.132*	
C2	0.7657 (9)	1.1427 (7)	0.7496 (4)	0.077 (2)	
H2B	0.8596	1.1213	0.7557	0.116*	
H2C	0.7119	1.0853	0.7792	0.116*	
H2D	0.7505	1.2344	0.7628	0.116*	
C3	0.7266 (6)	1.1227 (6)	0.6731 (3)	0.0476 (13)	
H3B	0.7820	1.1812	0.6431	0.057*	
C4	0.7495 (6)	0.9773 (5)	0.6511 (3)	0.0407 (12)	
C5	0.7091 (5)	0.7596 (6)	0.6763 (3)	0.0414 (12)	
C6	0.8381 (5)	0.8018 (5)	0.5786 (3)	0.0384 (11)	
C7	0.8293 (5)	0.9380 (5)	0.5933 (3)	0.0409 (12)	
C8	0.9025 (6)	1.0407 (6)	0.5488 (3)	0.0475 (13)	
C9	1.1203 (7)	1.0930 (8)	0.5012 (4)	0.082 (2)	
H9A	1.2130	1.0661	0.5064	0.123*	
H9B	1.1119	1.1866	0.5118	0.123*	
H9C	1.0916	1.0770	0.4532	0.123*	
C10	0.6696 (6)	0.7803 (7)	0.8495 (3)	0.0590 (17)	
H10A	0.6214	0.8067	0.8915	0.088*	
H10B	0.7080	0.8581	0.8272	0.088*	
H10C	0.7407	0.7193	0.8621	0.088*	
C11	0.6988 (7)	0.5213 (5)	0.7138 (4)	0.0561 (16)	
H11A	0.7529	0.5111	0.6717	0.084*	
H11B	0.6211	0.4640	0.7107	0.084*	
H11C	0.7515	0.4974	0.7547	0.084*	
C12	0.9120 (6)	0.7448 (6)	0.5171 (3)	0.0448 (13)	
C13	0.9775 (6)	0.6210 (6)	0.5252 (3)	0.0517 (14)	
H13A	0.9724	0.5762	0.5684	0.062*	
C14	1.0499 (7)	0.5646 (7)	0.4695 (3)	0.0630 (17)	
H14A	1.0974	0.4851	0.4756	0.076*	
C15	1.0497 (7)	0.6284 (7)	0.4061 (4)	0.0628 (18)	
C16	0.9852 (7)	0.7473 (7)	0.3944 (3)	0.0601 (17)	
H16A	0.9877	0.7878	0.3499	0.072*	
C17	0.9152 (6)	0.8071 (7)	0.4507 (3)	0.0534 (15)	
H17A	0.8709	0.8882	0.4438	0.064*	

### Atomic displacement parameters (Å^2^)

**Table:**

	*U*^11^	*U*^22^	*U*^33^	*U*^12^	*U*^13^	*U*^23^
S	0.0363 (6)	0.0583 (8)	0.0528 (8)	−0.0070 (7)	0.0064 (7)	0.0028 (8)
O1	0.079 (3)	0.059 (3)	0.077 (3)	0.018 (3)	0.013 (3)	0.031 (2)
O2	0.050 (2)	0.082 (3)	0.051 (2)	−0.014 (2)	0.006 (2)	0.012 (2)
O3	0.071 (3)	0.062 (3)	0.086 (3)	−0.019 (2)	0.018 (3)	0.009 (3)
O4	0.039 (2)	0.073 (3)	0.090 (4)	0.012 (2)	0.006 (2)	0.012 (3)
N1	0.045 (3)	0.046 (3)	0.048 (3)	0.002 (2)	0.004 (2)	−0.003 (2)
N2	0.043 (2)	0.031 (2)	0.045 (2)	0.0016 (19)	−0.005 (2)	−0.001 (2)
N3	0.048 (2)	0.038 (2)	0.041 (2)	0.000 (2)	0.002 (2)	0.005 (2)
F	0.117 (4)	0.110 (3)	0.076 (3)	0.019 (3)	0.038 (3)	−0.036 (3)
C1	0.072 (5)	0.048 (4)	0.143 (8)	0.024 (4)	−0.022 (5)	−0.020 (5)
C2	0.114 (7)	0.048 (4)	0.070 (4)	0.011 (4)	−0.024 (5)	−0.012 (3)
C3	0.049 (3)	0.040 (3)	0.054 (3)	−0.001 (3)	0.002 (3)	0.000 (3)
C4	0.040 (3)	0.042 (3)	0.040 (3)	−0.001 (2)	−0.001 (2)	0.005 (2)
C5	0.033 (3)	0.047 (3)	0.044 (3)	0.006 (2)	−0.001 (2)	0.007 (3)
C6	0.035 (3)	0.043 (3)	0.038 (3)	0.011 (2)	−0.004 (2)	0.003 (2)
C7	0.035 (3)	0.044 (3)	0.044 (3)	0.003 (3)	−0.005 (2)	0.007 (2)
C8	0.049 (3)	0.047 (3)	0.047 (3)	0.002 (3)	0.001 (3)	0.001 (3)
C9	0.064 (5)	0.110 (7)	0.072 (5)	−0.033 (5)	0.014 (4)	0.021 (5)
C10	0.056 (4)	0.082 (5)	0.039 (3)	−0.011 (3)	0.004 (3)	−0.005 (3)
C11	0.072 (4)	0.032 (3)	0.064 (4)	0.004 (3)	0.001 (3)	−0.001 (3)
C12	0.050 (3)	0.041 (3)	0.043 (3)	0.008 (3)	0.001 (3)	−0.007 (3)
C13	0.053 (3)	0.052 (3)	0.051 (3)	0.003 (3)	0.007 (3)	−0.003 (3)
C14	0.064 (4)	0.066 (4)	0.059 (4)	0.006 (4)	0.014 (3)	−0.005 (3)
C15	0.062 (4)	0.071 (4)	0.055 (4)	−0.003 (4)	0.014 (3)	−0.017 (4)
C16	0.067 (4)	0.069 (4)	0.044 (3)	0.004 (4)	0.012 (3)	−0.003 (3)
C17	0.053 (4)	0.064 (4)	0.044 (3)	0.010 (3)	0.005 (3)	−0.003 (3)

### Geometric parameters (Å, °)

**Table:**

S—O3	1.428 (4)	C4—C7	1.399 (7)
S—O4	1.429 (4)	C6—C7	1.392 (7)
S—N3	1.677 (5)	C6—C12	1.481 (7)
S—C10	1.738 (6)	C7—C8	1.509 (8)
O1—C8	1.187 (7)	C9—H9A	0.9600
O2—C8	1.348 (7)	C9—H9B	0.9600
O2—C9	1.446 (7)	C9—H9C	0.9600
N1—C4	1.329 (7)	C10—H10A	0.9600
N1—C5	1.324 (7)	C10—H10B	0.9600
N2—C5	1.335 (7)	C10—H10C	0.9600
N2—C6	1.345 (6)	C11—H11A	0.9600
N3—C5	1.384 (7)	C11—H11B	0.9600
N3—C11	1.483 (6)	C11—H11C	0.9600
F—C15	1.346 (7)	C12—C13	1.404 (8)
C1—C3	1.538 (9)	C12—C17	1.398 (8)
C1—H1B	0.9600	C13—C14	1.389 (8)
C1—H1C	0.9600	C13—H13A	0.9300
C1—H1D	0.9600	C14—C15	1.353 (9)
C2—C3	1.505 (9)	C14—H14A	0.9300
C2—H2B	0.9600	C15—C16	1.366 (9)
C2—H2C	0.9600	C16—C17	1.398 (8)
C2—H2D	0.9600	C16—H16A	0.9300
C3—C4	1.526 (8)	C17—H17A	0.9300
C3—H3B	0.9800		
			
O4—S—O3	119.2 (3)	C4—C7—C8	120.7 (5)
O4—S—N3	109.0 (3)	O1—C8—O2	124.0 (6)
O3—S—N3	105.6 (3)	O1—C8—C7	125.8 (6)
O4—S—C10	109.7 (3)	O2—C8—C7	110.2 (5)
O3—S—C10	107.6 (3)	O2—C9—H9A	109.5
N3—S—C10	104.9 (3)	O2—C9—H9B	109.5
C8—O2—C9	117.6 (5)	H9A—C9—H9B	109.5
C5—N1—C4	117.0 (5)	O2—C9—H9C	109.5
C5—N2—C6	116.4 (4)	H9A—C9—H9C	109.5
C5—N3—C11	120.1 (5)	H9B—C9—H9C	109.5
C5—N3—S	121.8 (4)	S—C10—H10A	109.5
C11—N3—S	117.2 (4)	S—C10—H10B	109.5
C3—C1—H1B	109.5	H10A—C10—H10B	109.5
C3—C1—H1C	109.5	S—C10—H10C	109.5
H1B—C1—H1C	109.5	H10A—C10—H10C	109.5
C3—C1—H1D	109.5	H10B—C10—H10C	109.5
H1B—C1—H1D	109.5	N3—C11—H11A	109.5
H1C—C1—H1D	109.5	N3—C11—H11B	109.5
C3—C2—H2B	109.5	H11A—C11—H11B	109.5
C3—C2—H2C	109.5	N3—C11—H11C	109.5
H2B—C2—H2C	109.5	H11A—C11—H11C	109.5
C3—C2—H2D	109.5	H11B—C11—H11C	109.5
H2B—C2—H2D	109.5	C17—C12—C13	118.6 (5)
H2C—C2—H2D	109.5	C17—C12—C6	122.6 (5)
C2—C3—C4	110.4 (5)	C13—C12—C6	118.8 (5)
C2—C3—C1	111.0 (6)	C14—C13—C12	120.9 (6)
C4—C3—C1	108.0 (5)	C14—C13—H13A	119.6
C2—C3—H3B	109.1	C12—C13—H13A	119.6
C4—C3—H3B	109.1	C15—C14—C13	118.3 (7)
C1—C3—H3B	109.1	C15—C14—H14A	120.8
N1—C4—C7	121.0 (5)	C13—C14—H14A	120.8
N1—C4—C3	114.8 (5)	F—C15—C14	117.8 (6)
C7—C4—C3	124.2 (5)	F—C15—C16	118.7 (6)
N1—C5—N2	126.9 (5)	C14—C15—C16	123.5 (6)
N1—C5—N3	118.7 (5)	C15—C16—C17	118.8 (6)
N2—C5—N3	114.3 (5)	C15—C16—H16A	120.6
N2—C6—C7	120.8 (5)	C17—C16—H16A	120.6
N2—C6—C12	115.0 (5)	C12—C17—C16	119.9 (6)
C7—C6—C12	124.2 (5)	C12—C17—H17A	120.1
C6—C7—C4	117.7 (5)	C16—C17—H17A	120.1
C6—C7—C8	121.6 (5)		
			
O4—S—N3—C5	50.2 (5)	C3—C4—C7—C6	−178.4 (5)
O3—S—N3—C5	179.3 (4)	N1—C4—C7—C8	−176.9 (5)
C10—S—N3—C5	−67.2 (5)	C3—C4—C7—C8	1.4 (8)
O4—S—N3—C11	−141.0 (4)	N2—C6—C7—C4	−3.3 (7)
O3—S—N3—C11	−12.0 (5)	C12—C6—C7—C4	176.5 (5)
C10—S—N3—C11	101.5 (5)	N2—C6—C7—C8	176.9 (5)
C9—O2—C8—O1	−8.8 (9)	C12—C6—C7—C8	−3.3 (8)
C9—O2—C8—C7	171.1 (5)	N2—C6—C12—C17	143.1 (6)
C5—N1—C4—C7	−0.2 (8)	C7—C6—C12—C17	−36.7 (8)
C5—N1—C4—C3	−178.7 (5)	N2—C6—C12—C13	−34.7 (7)
C4—N1—C5—N2	−3.3 (8)	C7—C6—C12—C13	145.5 (6)
C4—N1—C5—N3	176.9 (5)	C6—C7—C8—O1	121.1 (7)
C6—N2—C5—N1	3.3 (8)	C4—C7—C8—O1	−58.6 (8)
C6—N2—C5—N3	−176.9 (4)	C6—C7—C8—O2	−58.8 (7)
C5—N2—C6—C7	0.3 (7)	C4—C7—C8—O2	121.5 (6)
C5—N2—C6—C12	−179.5 (4)	C17—C12—C13—C14	3.2 (9)
S—N3—C5—N1	2.5 (7)	C6—C12—C13—C14	−178.9 (6)
S—N3—C5—N2	−177.3 (4)	C13—C12—C17—C16	−1.3 (9)
C11—N3—C5—N1	−165.9 (5)	C6—C12—C17—C16	−179.1 (6)
C11—N3—C5—N2	14.2 (7)	C12—C13—C14—C15	−3.5 (10)
C2—C3—C4—N1	53.8 (7)	C13—C14—C15—F	−179.6 (6)
C1—C3—C4—N1	−67.7 (7)	C13—C14—C15—C16	1.9 (11)
C2—C3—C4—C7	−124.6 (6)	C14—C15—C16—C17	−0.1 (11)
C1—C3—C4—C7	113.9 (7)	F—C15—C16—C17	−178.6 (6)
N1—C4—C7—C6	3.3 (8)	C15—C16—C17—C12	−0.2 (10)

### Hydrogen-bond geometry (Å, °)

**Table:**

*D*—H···*A*	*D*—H	H···*A*	*D*···*A*	*D*—H···*A*
C3—H3B···O1	0.98	2.52	3.181 (8)	125
C10—H10B···N1	0.96	2.56	3.148 (7)	120
C11—H11A···N2	0.96	2.23	2.697 (7)	109
C11—H11C···F^i^	0.96	2.34	3.302 (8)	177

Symmetry codes: (i) −*x*+2, −*y*+1, *z*+1/2.
